# Targeting the Parkinson’s disease using sim (*Rhodomyrtus tomentosa*) fruit water extract

**DOI:** 10.1186/s12906-025-04844-8

**Published:** 2025-05-14

**Authors:** Ngo Binh Thao Nghi, Dao My Linh, Dang Thi Phuong Thao

**Affiliations:** 1 Department of Molecular and Environmental Biotechnology, Faculty of Biology and Biotechnology, University of Science, 227 Nguyen Van Cu St, Dist 5, Ho Chi Minh City, Vietnam; 2 Vietnam National University, 227 Nguyen Van Cu St, Dist 5, Ho Chi Minh City, Vietnam; 3 Laboratory of Molecular Biotechnology, University of Science, 227 Nguyen Van Cu St, Dist 5, Ho Chi Minh City, Vietnam

**Keywords:** Parkinson's disease, Oxidative stress, Sim fruit water extract, DUCH knockdown, Drosophila melanogaster

## Abstract

Parkinson's disease (PD) is a progressive nervous system disorder characterized by dysfunction or degeneration of dopaminergic neurons in the substantia nigra leading to motor symptoms. Parkinson's disease is more common in the elderly and severely reduces quality of life. There has been no cure for PD, the therapy is mainly based on symptomatic treatment. Because of the strong relationship between oxidative stress and PD, antioxidant compounds have been considered a novel therapy for PD treatment. However, long-term treatment with a compound may cause potential risks and adverse effects. Consequently, screening natural compounds with therapeutic properties has elicited significant interest, thereby contributing to the expansion of the pharmacological source for disease treatment.

Sim (*Rhodomyrtus tomentosa*) is one kind of shrub, that belongs to the Myrtaceae family and has been used for a long time in traditional medicine of many Asian countries. All parts of sim can be used as medicine such as berried sim for treatment of diarrhea, and dysentery; leaves for wound healing, and roots for stomach cure. Previous studies suggested that sim fruit is rich in fiber, minerals, antioxidants, and anti-neurotoxic compounds. Utilizing antioxidant compounds from natural plants for PD therapy recently attracted attention. Because of its advantages, sim fruit has been supposed to be a potential candidate for drug screening.

No scientific evidence has been reported proving sim fruit's PD treatment capacity. This study thus aimed to evaluate the potential of the sim fruit water extract (SFWE) for PD treatment using the dUCH knockdown Drosophila melanogaster model which owns hallmark PD symptoms such as dopaminergic neuron degeneration and locomotor dysfunction. Our results showed that SFWE has a strong antioxidant capacity with an IC50 value of 55.55 ± 2.012 µg/mL. As expected, when treated with SFWE at 1.25 mg/mL, ROS induced by knockdown of dUCH specifically at dopaminergic neurons (DA) were reduced. Notably, the degeneration of DA neurons on the PD fly model was ameliorated by sim fruit extract. Consequently, the movement-defected symptoms were also rescued when the PD fly model was fed with sim fruit extract. Interestingly, SFWE caused no significant difference in fruit flies' development and lifespan, suggesting it’s safe to use. Taken together, our results strongly demonstrated that sim fruit, with its high antioxidant content, induced the decline of oxidative stress, and relieved symptoms, which is considered a potential candidate for PD treatment.

**Supplementary Information:**

The online version contains supplementary material available at 10.1186/s12906-025-04844-8.

## Introduction

Parkinson’s disease (PD) is a progressive nervous system disorder that predominantly occurs in elders. PD was identified by the degeneration of dopaminergic neurons (DA) in the brain's substantia nigra (SN) region, consequently causing a deficiency of the neurotransmitter dopamine, which is important for movement control [1]. The hallmark symptoms of PD include bradykinesia, tremor, rigidity, and postural instability [2]. Several non-DA neurons may also be affected later, leading to other non-motor symptoms such as cognitive impairment, fatigue, autonomic dysfunction, sleep disorder, etc. [3]. PD does not directly result in death but extremely reduces a patient’s quality of life. Recently, no available therapies can completely cure it, they just mitigate the progression of the disease.

Both genetic and environmental factors involve the contribution of PD, but still unclear. Numerous underlying mechanisms including oxidative stress are strongly proposed. In particular, the accumulation of reactive oxygen species (ROS) was proved to induce the loss of DA neurons, leading to the formation of PD [3, 4], as well as elevating ROS levels was also observed in the majority of patients [5]. Oxidative stress thus was emphasized as an important molecular mechanism related to the disease, opening novel insight not only for research but also for pharmaceutical applications [6]. Supporting these ideals, antioxidants such as vitamin C, and curcumin, ursolic acid were treated on the PD model, consequently achieving high efficiency in rescuing the symptoms [7–9]. Recently, medicinal treatment has garnered increased attention, some natural plants containing high levels of antioxidant compounds were also proposed as potential novel therapies for PD treatment [10–12].

Parkinson’s disease is a movement-related pathology, animal models can replicate these motor features are frequently thus utilized. Recently, through toxin-induced or genetic modifications, several Parkinson’s models have been established that are possible to mimic hallmark symptoms of Parkinson's disease, thereby promoting the screening of potential drugs [13]. The *Drosophila melanogaster* model with many advantages such as a relatively short life span, rapid growth, and cheap growth in a laboratory is increasingly widely used in research. Moreover, 70% of genes relating to disease in humans also occur in fruit flies. They not only have numerous similar biological processes but also can mimic the basic behaviors of humans, suitable for neuroscience studies. In particular, fruit flies have a relatively complete nervous system with DA clusters, which can simulate most of the characteristics helping to study Parkinson's disease more accurately and conveniently [14, 15]. Due to their strengths, many fruit fly models of PD have been created by mutagenesis or toxins with different symptoms. According to the study by Hiep et. al, dUCH knocked down in dopaminergic neurons in fruit flies was able to mimic the phenotypes of PD including impaired mobility, loss of dopaminergic neurons [16]. This fly model was also used in several previous research to evaluate the therapeutic potential of antioxidant compounds and plant extract [11, 12]. Therefore, it is a promising model for Parkinson's disease drug screening.

Sim (*Rhodomyrtus* tomentosa) is a heliophilous shrub belonging to the *Myrtaceae* family. All parts of the sim have pharmaceutical value and could be used for traditional medicine in Asian countries [17]. Sim fruit is a kind of berry with an elliptical shape with a diameter from 1 to 1.5 cm, green when unripe, and brownish red when ripe. Berried sim was utilized in traditional medicine of several Southeast Asian countries, and China. In particular, sim fruit was used in the treatment of digestive and urinary problems such as dysentery, diarrhea, and infections [18, 19]. In Vietnam, local people believe that berried sim also improves the immune system, they thus are used not only as medicine but also as supplement food products [17, 20, 21]. Pharmaceutical properties of sim fruit such as anti-inflammatory, antioxidant, and anti-obesity are due to the high content of phenolic compounds. 19 antioxidant compounds contained in sim fruit were defined and quantified, in which piceatannol is the component with the highest concentration as well as pharmaceutical value [22]. Besides that, sim fruit is rich in fiber, minerals, and vitamins and low in sugar and fat, they thus are considered a low-calorie food [23]. As the above advantages, sim fruit can become a natural food source with many healthy ingredients, especially antioxidant properties.

The evidence discussed above raises interesting questions about the therapeutic efficacy of sim fruit water extract (SFWE) in the treatment of Parkinson’s disease. To examine this question, we aim to investigate the impact of SFWE on developmental processes and disease symptoms using the *Drosophila melanogaster* model.

## Material and methods

### Plant collection and preparation

Sim fruit (voucher specimen number: PHH1004933 identified by Dr. Dang Le Anh Tuan, Faculty of Biology and Biotechnology, University of Science, Ho Chi Minh City, Vietnam) was collected from Phu Quoc in July. Sim fruit was conducted following the protocol of Truong et.al [11] with some modifications. Sim fruits were washed with water, deep-frozen, and then freeze-dried. Then the dried fruit was ground into powder and dissolved in water with a ratio of 1: 8. The mixture was then incubated for 30 min and centrifuged for 20 min at 4°C. The supernatant was collected, continuously deep-frozen, and freeze-dried. Finally, the dry matter of sim fruit water extract (SFWE) was stored at −30°C and used for further experiments. SFWT avoided light, high temperature, and moisture to prevent the alternation of ingredients in the extract.

### Fly stock culture and generated

Fly stocks were maintained on the standard medium (5% dry yeast, 5% sucrose, 3% powdered milk, 0.1% sodium benzoate, 0.5% propionic acid, and 0.8% agar) and kept at 25°C.

For the experiment, flies were cultured on a standard medium and the standard medium was supplemented with different concentrations of sim fruit water extract (SFWE) and kept at 28°C. The medium was avoided light and high temperature for good quality.

Tyrosine hydroxylase (TH) is an enzyme that plays a crucial role in the synthesis of dopamine, it is thus used as the driver for dopaminergic neurons. TH-GAL4 driver line (Bloomington Drosophila stock center, code #8848) specifically expressed Gal4 protein at the dopaminergic neurons, while the UAS- RNA interference line (Vienna Drosophila Resource Center, code #v26468) carried UAS- dUCHIR sequence. The knockdown of dUCH in dopaminergic neurons was achieved by crossing the TH-Gal4 driver with UAS-dUCHIR, thereby establishing a *Drosophila* model for Parkinson’s disease. The control was generated by crossing the TH-Gal4 driver with another UAS- RNA interference line (Bloomington Stock Center, code #9331) carrying UAS-GFP-IR sequence. GFP did not exist in fruit flies, so the control had a similar background to knockdown flies.

### DPPH (1,1-diphenyl-2-picrylhydrazyl) assay

The antioxidant activity was evaluated based on the discoloration of DPPH (1,1-diphenyl-2-picrylhydrazyl) when associated with antioxidants. The protocol followed the study of Truong et.al [11] with some modifications. The antioxidant capacity standard curve of SFWE was performed by 6 concentrations from 12.5 μg/mL to 400 μg/mL. The standard curve of Vitamin C (L-ascorbic acid -#A0278-25G, Sigma, Singapore) was performed by concentrations from 5 μM to 25 μM utilized as standard control. DPPH reaction consisted of different concentrations of the extract or standard compound, methanol solvent, and DPPH reagent (#D9132, Sigma, Singapore), meanwhile, the blank contained methanol and distilled water; was incubated at 30°C for 40 min and avoided light. The negative control containing methanol, DPPH, and distilled water was also done. The spectrometer then recorded the amount of the rest of the DPPH at 517 nm absorbance. Raw data was recorded in Microsoft Excel and calculated following the formula below to receive the DPPH radical scavenging activity. The half-maximal inhibitory concentration (IC50). Statistical tests were performed with GraphPad 8.0.2 [24].

$$\text{DPPH radical scavenging acitivity}=100-\frac{\text{Abs sample}-\text{Abs blank sample}}{\text{Abs negative control}}*100$$

### Toxicity and development assay

Embryos were embedded into experimental media containing SFWE at various concentrations (70 embryos/vial). All vials would then be cultured at 28°C. The number of newly formed pupae (yellow pupae) and newly eclosed flies were observed and recorded every day. The raw data was recorded in Microsoft Excel. Statistical tests were performed with GraphPad 8.0.2.

### Life span assay

The newly eclosed male flies were collected and transferred into new vials containing experimental medium prepared the day prior. For 2–3 days, flies were transferred into new vials; at the same time, the number of fly deaths was noted every day until all flies died. Data was recorded in Microsoft Excel. Statistical tests were performed with GraphPad 8.0.2.

### Food intake assay

The assay following the protocol of Thoa et.al with some modification [11]. The food intake medium containing 2% Coomassie Brilliant Blue G-250 (#808274–10 g, Biomedicals, USA), standard medium, and SFWE at different concentrations, which was prepared in a 1.5 mL microcentrifuge tube. Early third-instar larvae were selected randomly and transferred into tubes within 30 min. Then larvae were transferred into new tubes (10 larvae per tube), ground in PBS-10% ethanol, and centrifuged for 10 min. The supernatant was received and measured by an optical density (OD) level of 595 nm. Raw data were collected by Microsoft Excel 2016 (Microsoft, USA). Statistical tests were performed with GraphPad 8.0.2.

### Climbing assay

The assay following the protocol of Thoa et.al with some modification [25]. The male flies in each train were randomly selected after eclosed and maintained at 28°C with 20 flies per vial. For every 2 days, flies were transferred into new vials with a medium containing different extract concentrations. The assay was performed every 10 days, from 1 to 30 days old. On the experimental day, flies were transferred into climbing vitals (height of 20 cm, diameter of 2 cm) and acquainted for one hour. To begin at the same starting point, the climbing vital was tapped five times and flies would be climbed up freely in 60 s, repeated 5 times. This process was recorded by digital camera and the height of the fly after 5 s was marked as follows: 0 (< 2 cm), 1 (2–4 cm), 2 (4–6 cm), 3 (6–8 cm), 7 (14–16 cm), and 8 (more than 16 cm). Data was recorded in Microsoft Excel. Statistical tests were performed with GraphPad 8.0.2

### Immunofluorescent

The experiment followed the protocol of Hiep et. al with some modifications [16]. The brains of larvae/ flies were dissected and kept in cold PBS. The samples were fixed in PBS containing 4% PFA for 20 min. Afterward, the samples were washed with PBS-T 0.3% quickly 2 times and with a rocker for 20 min 2 times. Next, the samples were placed in blocking solution (consisting of 10% NGS, 0.15% PBS-T in PBS) for 20 min at 25°C, after which we added anti- Tyrosine hydroxylase antibody (anti-TH; Millipore, AB152, Japan) at a 1:250 ratio. The samples were then incubated for 36 h at 4°C. Afterward, the samples were continuously quickly washed with PBS-T 0.3% 2 times and with a laboratory rocker 5 times for 20 min each. The blocking step was repeated as described above, after which the samples were incubated with a second antibody Alexa 488 (1: 500; Invitrogen) at a 1:500 ratio for 2 h. Following the incubation, we repeated the washing step as described previously. Finally, the samples were mounted in Vectashield Mounting Medium, observed the images with an ECLIPSE Ni-U fluorescence microscope (Nikon). Raw images were visualized and analyzed by ImageJ. Statistical test was performed with GraphPad 8.0.2.

### ROS assay

Brains of male 3rd instar larvae were dissected in phosphate-buffered saline (PBS) and then incubated in 10 uM CM-H_2_DCFDA (C6827; Invitrogen) for 10 min, avoiding light. The samples then were washed with PBS three times and immediately mounted in Vectashield Mounting Medium (Vector Laboratories, Tokyo, Japan). The samples were observed by an ECLIPSE Ni-U fluorescence microscope (Nikon). Raw images were visualized and analyzed by ImageJ. Statistical test was performed with GraphPad 8.0.2.

### Statistical test

Raw data were collected using Microsoft Excel (Microsoft) and statistically analyzed by GraphPad Prism 8.0.2 (GraphPad Software). Parametric oneway-ANOVA with Sidak multiple comparison test was applied for analyzing raw data results of toxicity, development, food intake, immunofluorescence, and ROS experiments. For survival assay and climbing assay, the Mantel-Cox and Kurkal Wallis Dunn’s multiple comparison test were utilized, respectively.

## Result

### Antioxidant capability of sim fruit water extract

**Fig. 1:**
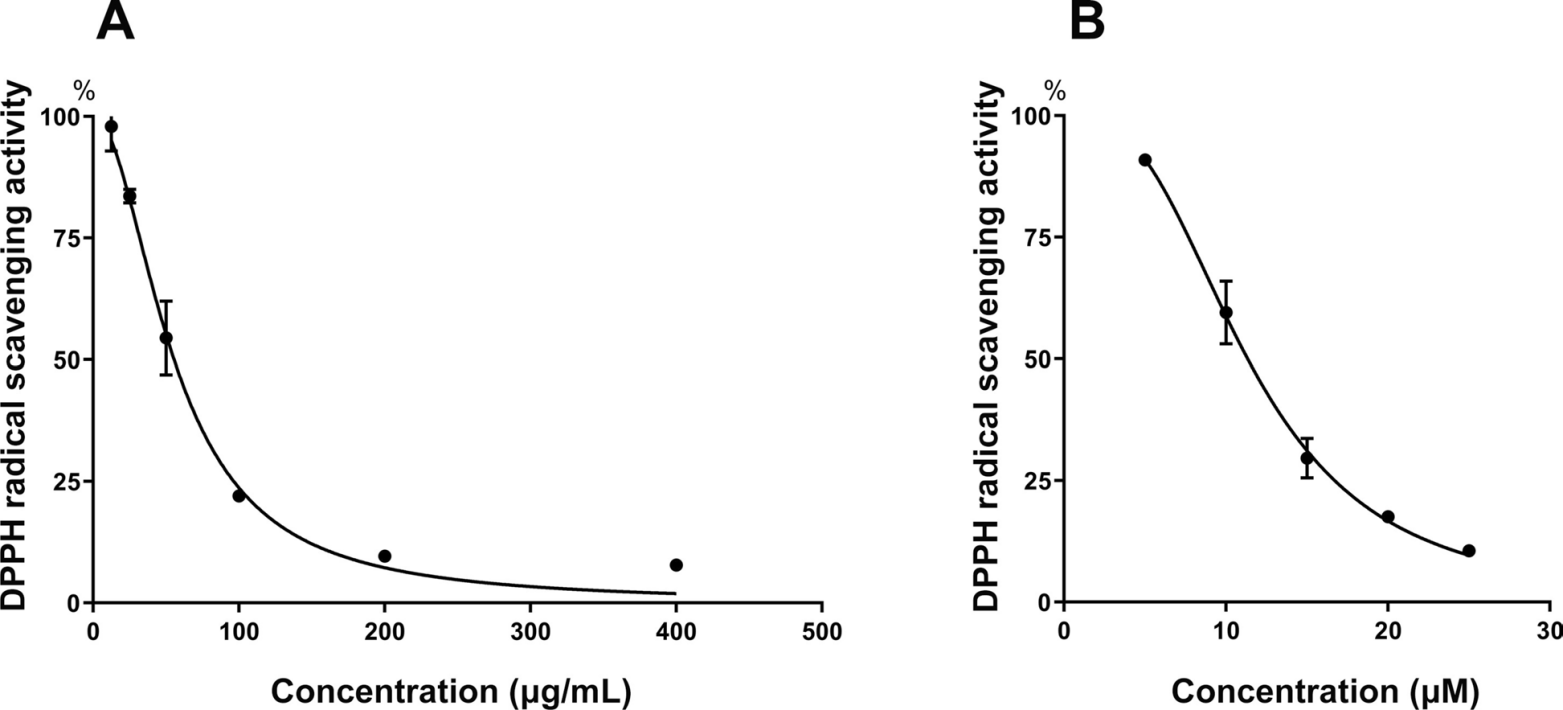
The antioxidant capacity of sim fruit water extract (SFWE). **A** DPPH radical scavenging activity of SFWE, *R*^2^ = 0.9856, IC_50_ = 55.55 ± 2.012 µg/mL. **B** DPPH radical scavenging activity of vitamin C, *R*.^2^ = 0.9873, IC_50_ = 11.34 ± 0.235 µM. Data represent means and the standard deviation (SD)

The antioxidant capability of sim fruit water extract (SFWE) was estimated by DPPH radical scavenging activity assay; simultaneously the standard compound used was vitamin C. The result showed that the IC_50_ value of SFWE was 55.55 ± 2.012 µg/mL (Fig. 1-A, *R*^2^ = 0.9856), equivalent to an antioxidant capacity of 11.34 ± 0.235 µM vitamin C (Fig. 1-B, *R*^2^ = 0.9873). Previous research suggested that an antioxidant capacity of vitamin C at 0.5 µM, equivalent to 2.5 mg/mL SFWE, would most effectively rescue the PD-like phenotype [7]. We thus decided to use SFWE at concentrations of 1.25, 2.5, and 5 mg/mL for further experiments on fruit flies.

### Impact of sim fruit water extract on *Drosophila melanogaster* development

**Fig. 2:**
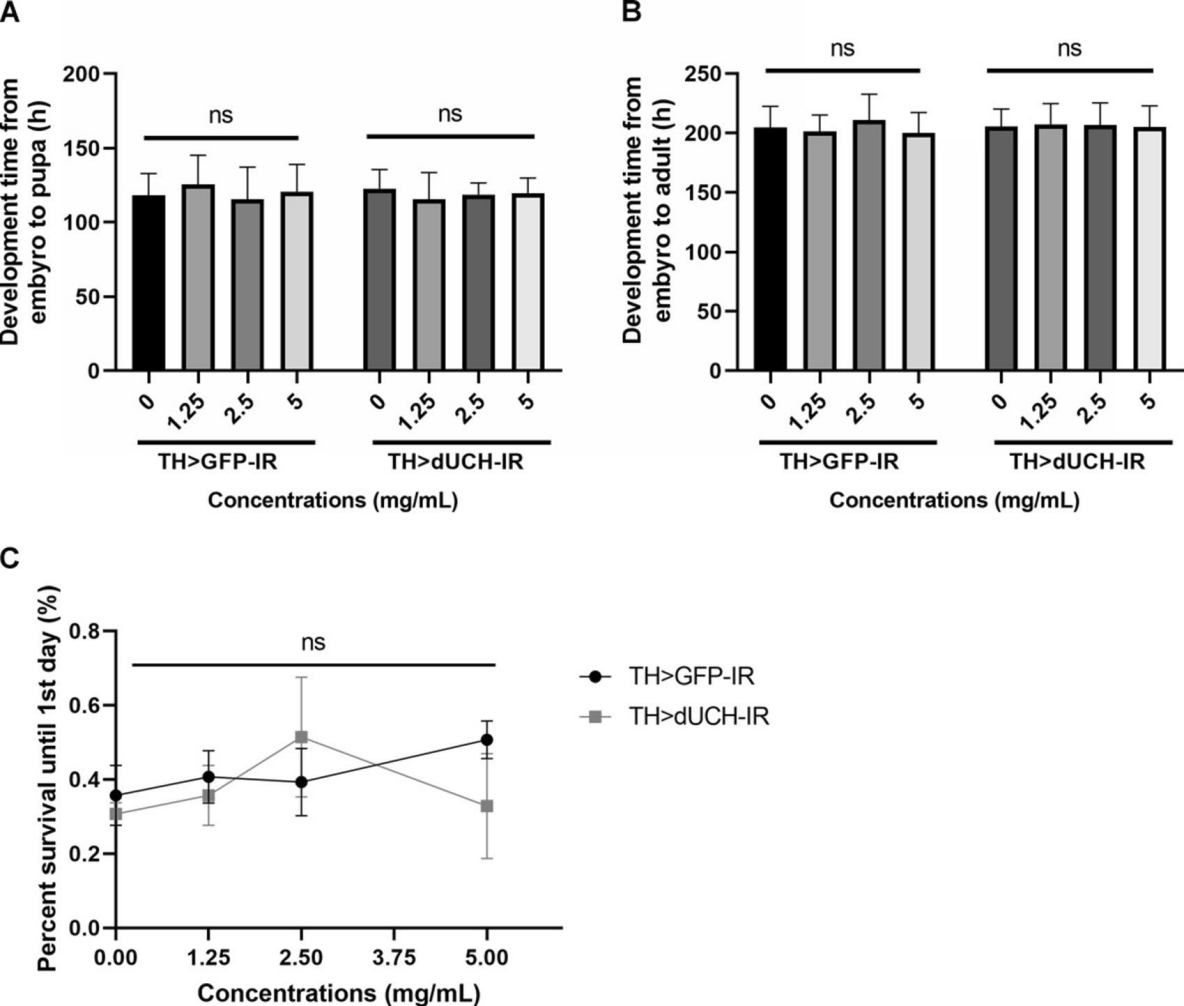
Effects of sim fruit water extract (SFWE) on *Drosophila melanogaster* physiology. **A** Development time from embryo to pupa. **B** Development time from embryo to adult. Sample size *n* = 70 embryos per strain; one-way ANOVA, Sidak multiple comparison test. **C** Percent survival of fruit flies from embryos to adult flies 1 day old. Biological replicate *n* = 2, each containing 70 embryos per strain; two-way ANOVA. The control strain TH > GFP-IR (+ ; UAS-GFP-IR; TH-GAL4) and dUCH-knockdown strain TH > dUCH-IR (+ ; + ; TH-GAL4/UAS-dUCH-IR), ns: not significant. Data represents means and the standard deviation (SD)

It is known that overuse of antioxidant compounds may lead to some unexpected effects [26, 27]. Since abnormal growth and metamorphosis can reflect toxicity, we first investigated the development time of flies when treated with the SFWE. Our data showed that treating with SFWE at all investigated concentrations did not alternate the development time of dUCH knockdown flies in both 2 periods including embryo to pupa stage (Fig. 2-A, *p* > 0.05, one-way ANOVA, Sidak multiple comparison test) and embryo to adult stage (Fig. 2-B, *p* > 0.05, one-way ANOVA, Sidak multiple comparison test). Similarly, in control flies, the effect on growing time was not reported (Fig. 2-A, B, *p* > 0.05, one-way ANOVA, Sidak multiple comparison test). Besides growing time, the percentage survival of fruit flies from embryos to adult flies 1 day old also suggested the toxicity of SFWE in the early stage. Consistent with previous results, the percent survival of both fly trains was not significantly affected by SFWE at all treated concentrations (Fig. 2-C, *p* > 0.05, two-way ANOVA).

### Sim fruit water extract effect on lifespans

**Fig. 3:**
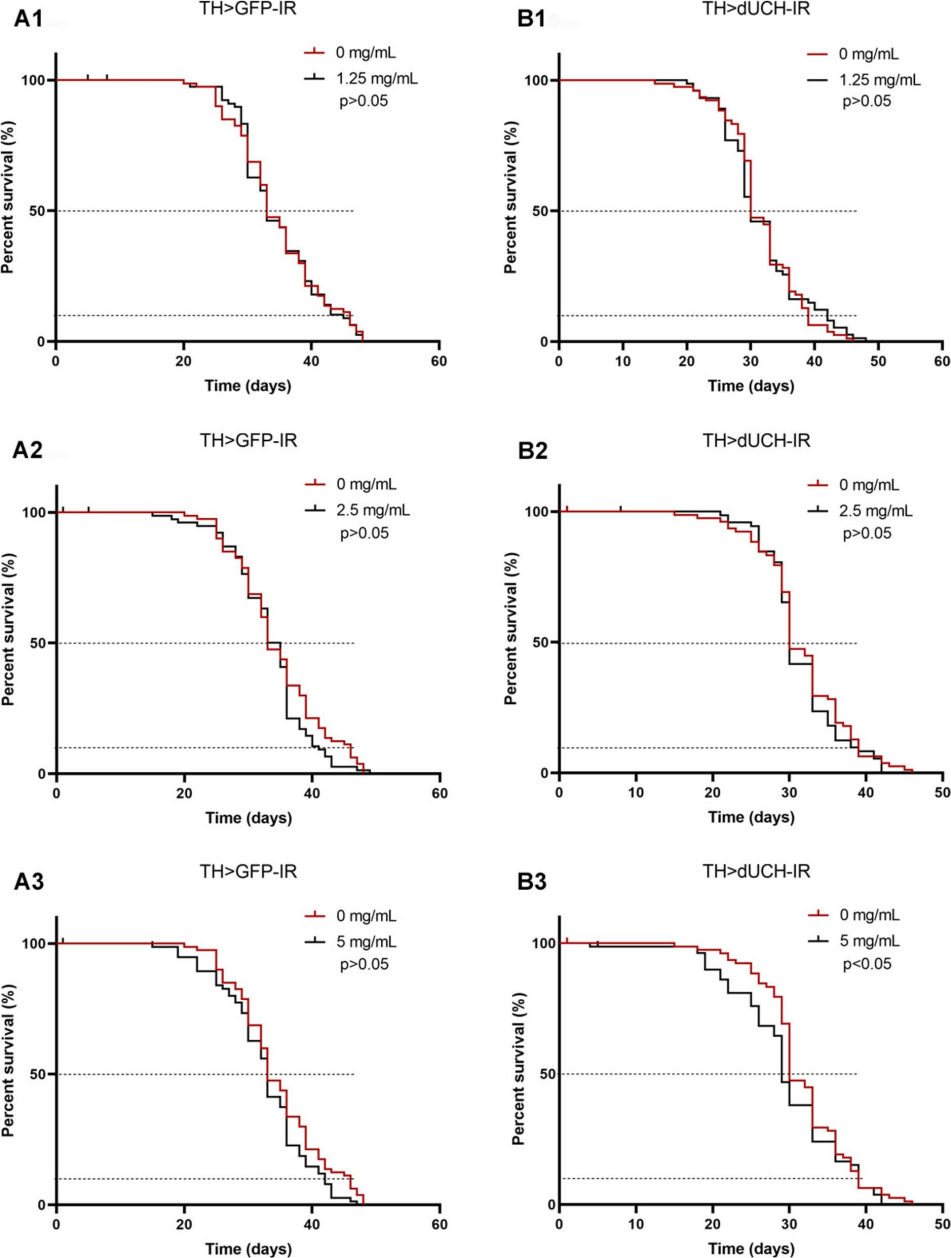
Effect of sim fruit water extract (SFWE) on *Drosophila melanogaster* lifespans. **A1**-**A3** The comparisons between concentrations from 1.25 to 5 mg/mL, separately, and 0 mg/mL on the control strain. **B1**-**B3** The comparisons between concentrations from 1.25 to 5 mg/mL, separately, and 0 mg/mL on the dUCH knockdown strain. Sample size *n*= 100 flies per train, Log-rank test The control strain TH>GFP-IR (+; UAS-GFP-IR; TH-GAL4) and dUCH-knockdown strain TH>dUCH-IR (+; +; TH-GAL4/UAS-dUCH-IR), ns: not significant, *p<0.05

Along with toxicity experiments, we also evaluated the lifespans of different SFWE concentrations to determine their accumulative effect. Our data demonstrated that SFWE did not change the vitality of control flies at all investigated concentrations (Fig. 3-A1-A4, *p*>0.05, Log-rank test) while the survival rate of dUCH-knockdown flies declined when using SFWE at high concentrations, concretely 5 mg/mL (Fig. 3-B3, *p*<0.05, Log-rank test). Moreover, correlating with the previous reports, dUCH knockdown flies showed shortened lifespans compared to the non-treated control (Fig. S1, *p*<0.05, Log-rank test) [7, 11, 12].

### Sim fruit water extract altered the food intake of normal flies but not defective flies

**Fig. 4:**
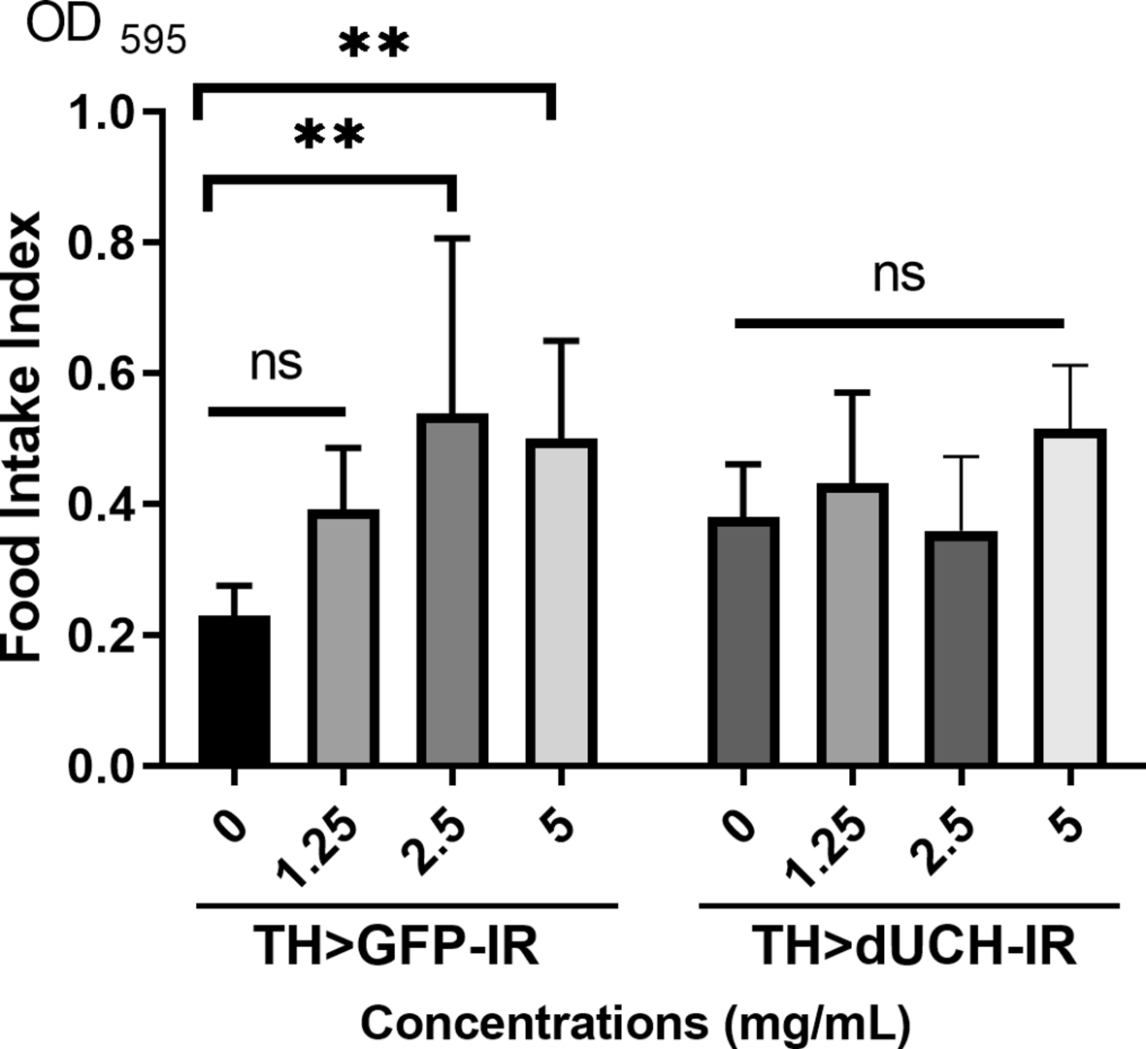
Effect of sim fruit water extract (SFWE) on *Drosophila melanogaster *food intake. Biological replicate *n*= 6, each containing 10 larvae per strain, one-way ANOVA, Sidak multiple comparison test. The control strain TH>GFP-IR (+; UAS-GFP-IR; TH-GAL4) and dUCH-knockdown strain TH>dUCH-IR (+; +; TH-GAL4/UAS-dUCH-IR), ns: not significant, **p<0.01, ***p<0.001. Data represents means and the standard deviation (SD)

**Fig. 5:**
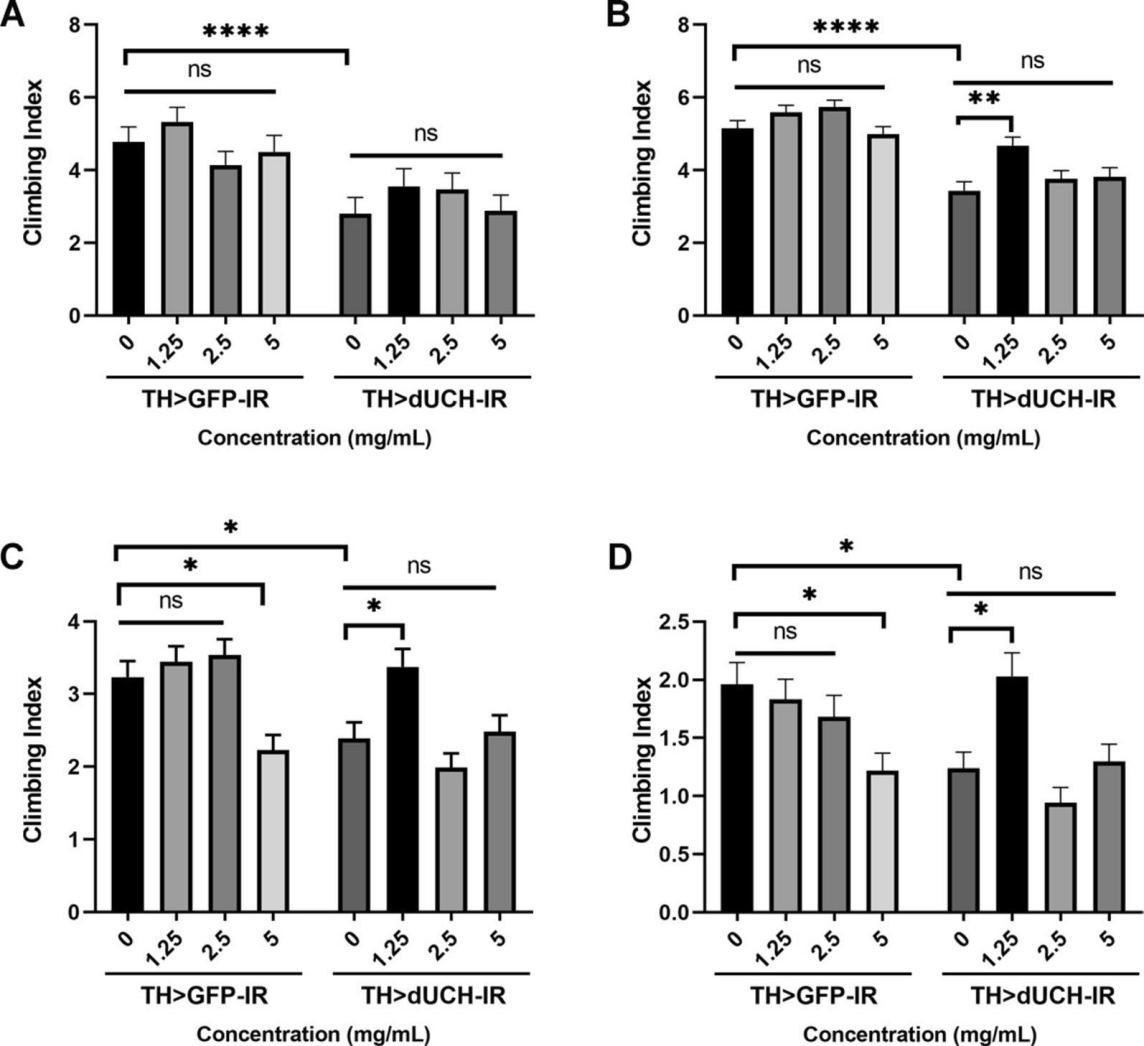
Effect of sim fruit water extract (SFWE) on *Drosophila melanogaster* locomotor symptom. The extract at 1.25 mg/mL concentration rescued the climbing ability of dUCH knockdown flies from 10 days old to 30 days old. **A**: 1 day old. **B**: 10 days old. **C**: 20 days old: **D**: 30 days old. Sample size *n* = 80 flies per strain, Kruskal- Wallis, Dunn’s multiple comparison test. The control strain TH > GFP-IR (+ ; UAS-GFP-IR; TH-GAL4) and dUCH-knockdown strain TH > dUCH-IR (+ ; + ; TH-GAL4/UAS-dUCH-IR), ns: not significant, *p < 0.05, **p < 0.01, and ****p < 0.0001. Data represents means and the standard error (SE)

In this study, fruit flies were treated with SFWE by oral route, the extract supplement may cause changes in the medium’s flavor, the amount of food intake thus was an important factor that should be reported to prevent misjudgment. The feeding assay was performed to investigate the amount of received food in both fly strains at 0, 1.25, 2.5, and 5 mg/mL concentrations. Experimental data showed that SFWE did not significantly alternate the food intake in dUCH knockdown flies at all investigated concentrations (Fig. 4, *p* > 0.05, one-way ANOVA, Sidak multiple comparison test. On the other hand, in the control flies, significant differences were reported at 2.5 mg/mL and 5 mg/mL concentrations (Fig. 5, *p* < 0.01, one-way ANOVA, Sidak multiple comparison test). Taken together, SFWE extract affected the food intake of control flies, but not in all investigated concentrations.

### Sim fruit water extract rehabilitated locomotor dysfunction of dUCH knockdown *Drosophila*

As described above, movement impairment is a main characteristic symptom of Parkinson's disease. Following this conjecture, we first conducted experiments to evaluate the effects of SEWE at 0, 1.25, 2.5, and 5 mg/mL concentrations on the locomotor ability of adult flies.

**Fig. 6:**
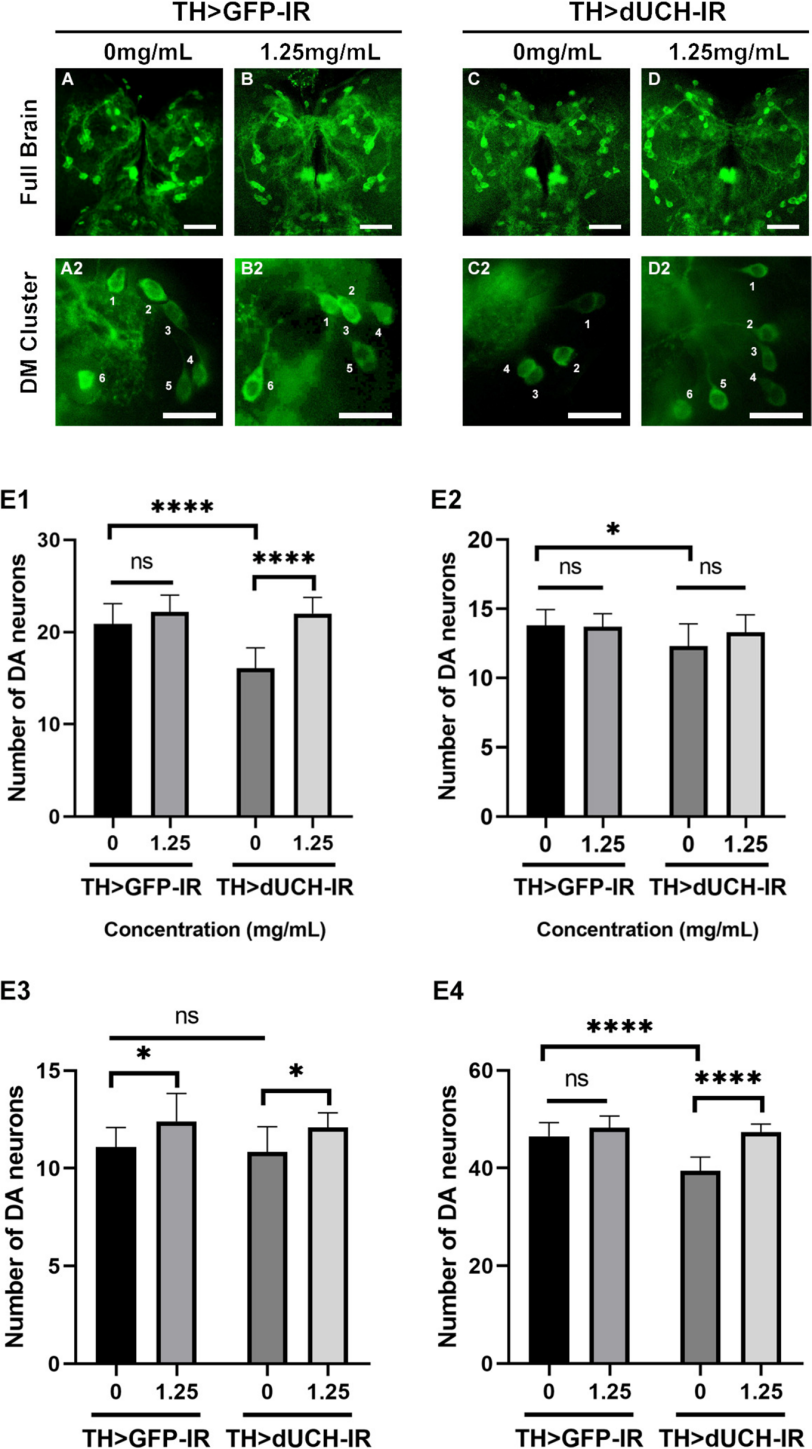
Sim fruit water extract rescued the loss of dopaminergic neurons (DA) caused by the knockdown of dUCH in larvae. **A**-**D** Immunostaining image of full brain lobes with anti-tyrosine hydroxylase antibody, scale bars indicate 20 μm. A1-D1: Immunostaining image of DM cluster, scale bars indicate 5 μm. E1: The data described the number of DA neurons in DM clusters. E2: The data described the number of DA neurons in DL1 clusters. E3: The data described the number of DA neurons in DL2 clusters. E4: The data described the number of DA neurons in total clusters. Sample size *n* = 10–13 flies per strain, one-way ANOVA, Sidak multiple comparison test. The control strain TH > GFP-IR (+ ; UAS-GFP-IR; TH-GAL4) and dUCH-knockdown strain TH > dUCH-IR (+ ; + ; TH-GAL4/UAS-dUCH-IR). ns: not significant, *p < 0.05, and ****p < 0.0001. Data represents means and the standard deviation (SD)

Based on the result, the decrease in locomotor due to dUCH deficiency was reported in all examined days (Fig. 5-A-D, *p* < 0.0001, *p* < 0.0001, *p* < 0.01 and *p* < 0.05, respectively, Kruskal- Wallis, Dunn’s multiple comparison test). SFWE at the concentrations of 1.25 mg/mL rehabilitated the mobility defect of dUCH knockdown flies, compared to the untreated fly, which started from 10 days old to 30 days old (Fig. 5B-D, *p* < 0.01, *p* < 0.05, *p* < 0.05 respectively, Kruskal- Wallis, Dunn’s multiple comparison test). In detail, the climbing index increased by 36.2% when supplement extract at 10 days old, similarly, there were 41.3% and 63.7% at 20 days old and 30 days old. However, there was no improvement when flies were treated at other concentrations (Fig. 6A-D, *p* > 0.05, Kruskal- Wallis, Dunn’s multiple comparison test). By contrast, 1.25 mg/mL of SFWE did not significantly impact the climbing index of the control (Fig. 6A-D, *p* > 0.05, Kruskal- Wallis, Dunn’s multiple comparison test), meanwhile, the negative effect was reported at 5 mg/mL in the later stage (Fig. 6C-D, *p* < 0.01, *p* < 0.05 respectively, Kruskal- Wallis, Dunn’s multiple comparison test).

### Sim fruit water extract rescued the degeneration of dopaminergic neurons in *dUCH* knockdown fruit flies

The loss of dopaminergic neurons is a specific hallmark of Parkinson’s disease. Therefore, assessing dopaminergic neuron degeneration is an important outcome that demonstrates the therapeutic potential of SFWE. In this study, we examined the number of DA neurons through immunofluorescence with tyrosine hydroxylase antibody- an enzyme specific for the dopamine synthesis process. The larvae brain lope was divided into 3 clusters including DM, DL1, and Dl2, while there was PAL, PPL1, PPL2, PPM1/2, and PPM3 in adult flies [28]. The assay was performed at 2 concentrations including 0 mg/mL and 1.25 mg/mL because of its effectiveness in locomotor improvement.

Data showed that dUCH knockdown larvae had a severe loss of DA neurons in DM cluster (Fig. 6-B, *p* < 0.0001, one-way ANOVA, Sidak multiple comparison test), DL1 cluster (Fig. 6-C, *p* < 0.05, one-way ANOVA, Sidak multiple comparison test), and the total cluster (Fig. 6-E, *p* < 0.0001, one-way ANOVA, Sidak multiple comparison test), compared to control. Interstingly, the degeneration of dopaminergic (DA) neurons was significantly rescued by SFWE at the concentration of 1.25 mg/mL in DM cluster (Fig. 6-B, *p* < 0.001, one-way ANOVA, Sidak multiple comparison test), DL2 cluster (Fig. 6-D, *p* < 0.05, one-way ANOVA, Sidak multiple comparison test), and total DA neurons (Fig. 6-E, *p* < 0.0001, one-way ANOVA, Sidak multiple comparison test), meanwhile, there was no significant difference in the control strain (Fig. 6, *p* > 0.05, one-way ANOVA, Sidak multiple comparison test).

**Fig. 7:**
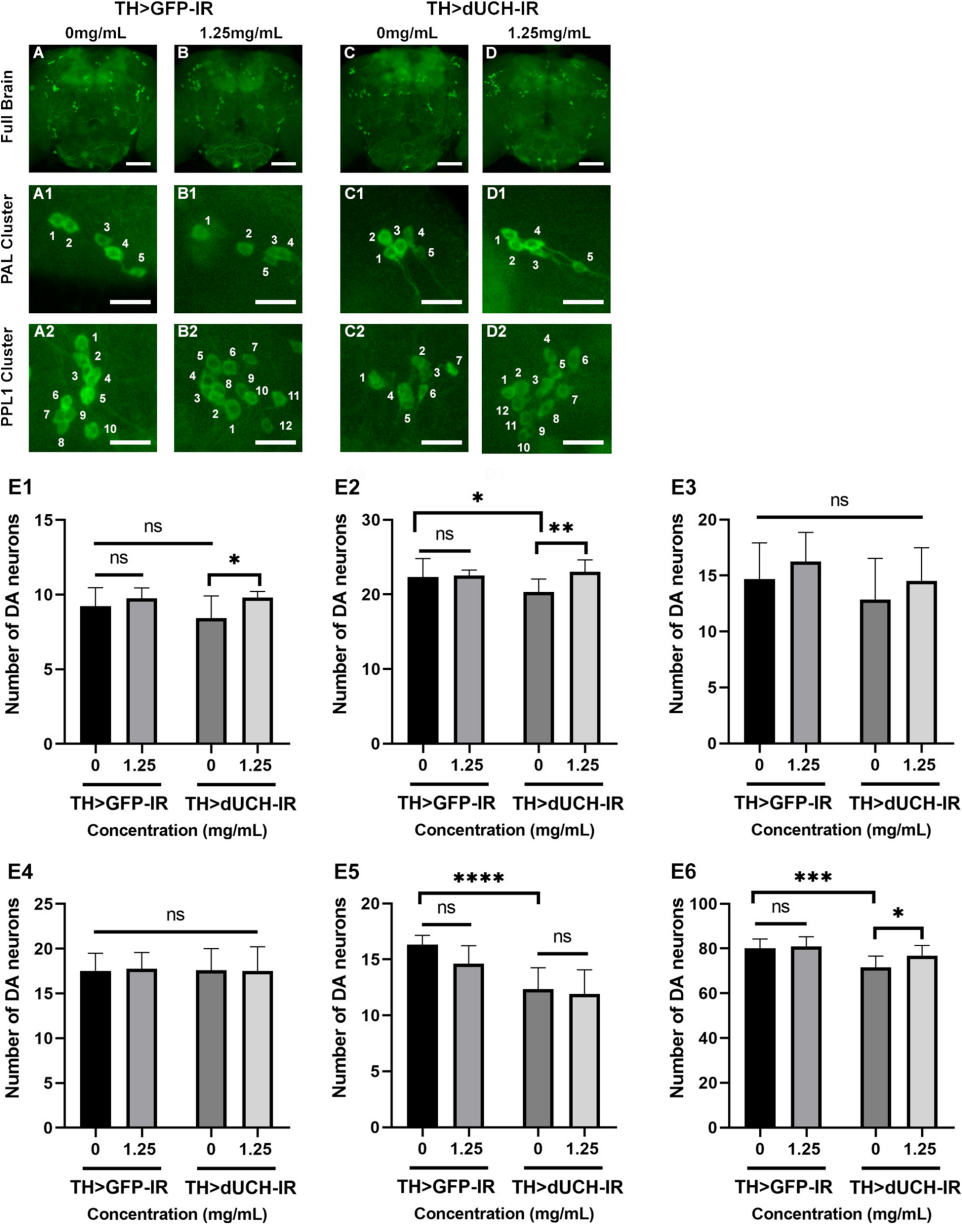
Sim fruit water extract rescued the loss of dopaminergic neurons (DA) caused by the knockdown of dUCH in adults 10 days old. **A**-**D** Immunostaining image of full brain with anti-tyrosine hydroxylase antibody, scale bars indicate 20 μm. A1-D1: Immunostaining image of PAL cluster, scale bars indicate 5 μm. A2-D2: Immunostaining image of PPL1 cluster, scale bars indicate 5 μm. E1: The data described the number of DA neurons in PAL clusters. E2: The data described the number of DA neurons in PPL1 clusters. E3: The data described the number of DA neurons in PPL2 clusters. E4: The data described the number of DA neurons in PPM1/2 clusters. E5: The data described the number of DA neurons in PPM3 clusters. E6: The data described the number of DA neurons in total clusters. Sample size *n*= 8-12 flies per strain, one-way ANOVA, Sidak multiple comparison test. The control strain TH>GFP-IR (+; UAS-GFP-IR; TH-GAL4) and dUCH-knockdown strain TH>dUCH-IR (+; +; TH-GAL4/UAS-dUCH-IR). ns: not significant, *p<0.05, **p<0.01, ***p<0.001 and ****p<0.0001. Data represents means and the standard deviation (SD)

**Fig. 8:**
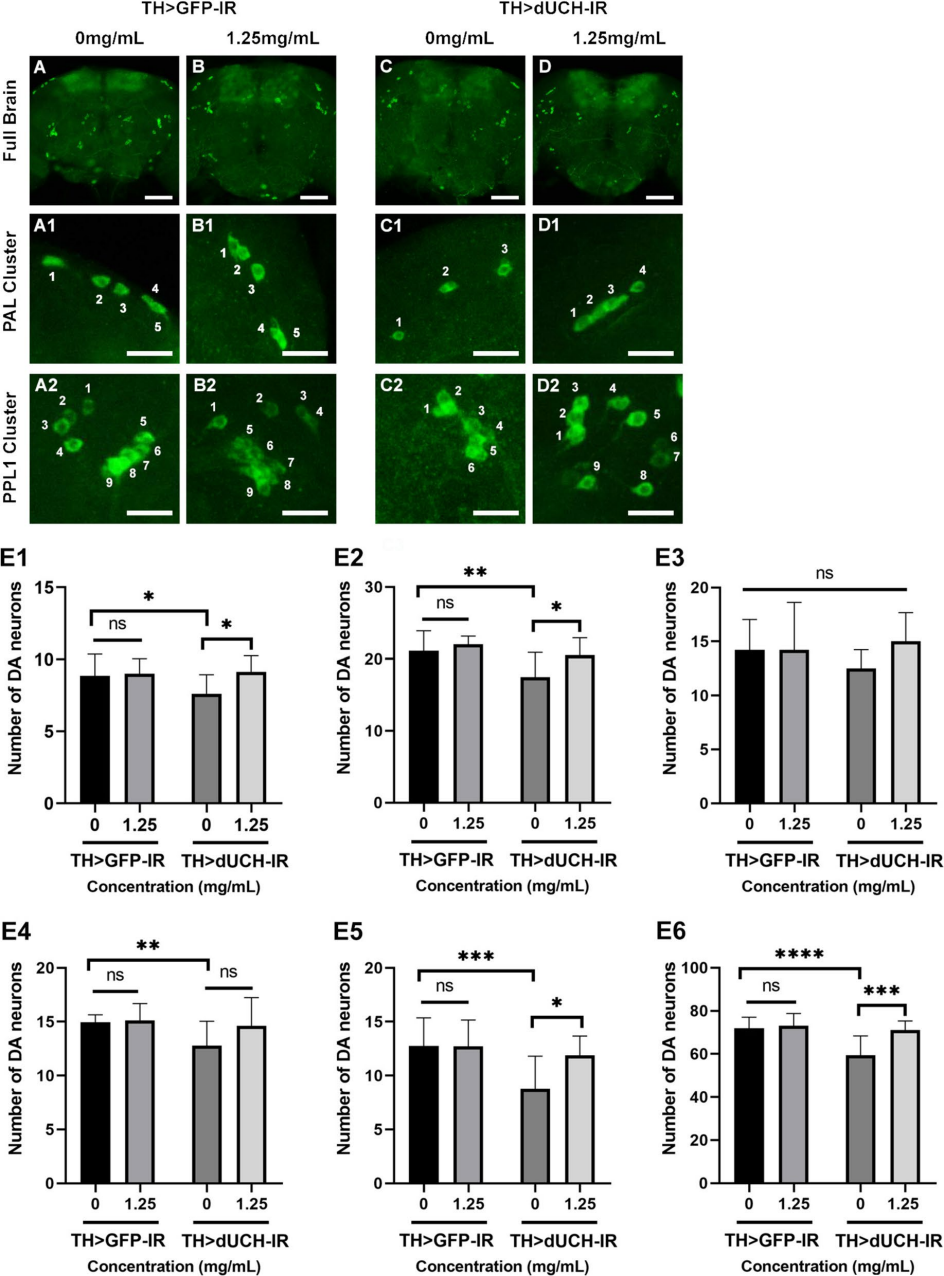
Sim fruit water extract rescued the loss of dopaminergic neurons (DA) caused by the knockdown of dUCH in adults 30 days old. **A**-**D** Immunostaining image of full brain with anti-tyrosine hydroxylase antibody, scale bars indicate 20 μm. A1-D1: Immunostaining image of PAL cluster, scale bars indicate 5 μm. A2-D2: Immunostaining image of PPL1 cluster, scale bars indicate 5 μm. E1: The data described the number of DA neurons in PAL clusters. E2: The data described the number of DA neurons in PPL1 clusters. E3: The data described the number of DA neurons in PPL2 clusters. E4: The data described the number of DA neurons in PPM1/2 clusters. E5: The data described the number of DA neurons in PPM3 clusters. E6: The data described the number of DA neurons in total clusters. Sample size *n*=10-15 flies per strain, one-way ANOVA, Sidak multiple comparison test. The control strain TH>GFP-IR (+; UAS-GFP-IR; TH-GAL4) and dUCH-knockdown strain TH>dUCH-IR (+; +; TH-GAL4/UAS-dUCH-IR). ns: not significant, *p<0.05, **p<0.01, ***p<0.001 and ****p<0.0001. Data represents means and the standard deviation (SD)

Previous studies suggested that the number of DA neurons is reduced over time due to not only the progression of PD but also the aging process [16]. We, therefore, continuously performed the assay at 1, 10, and 30 days old to further evaluate the long-term effectiveness of treatment. As expected, utilizing SFWE at a concentration of 1.25 mg/mL was able to delay the degradation of DA neurons in dUCH knockdown flies. The extract's effectiveness was noticed at 1 day old in the total number of DA neurons but not in individual clusters (Fig. S2, *p*<0.01, one-way ANOVA, Sidak multiple comparison test). The loss of neurons was continuously rescued at 10 days old, which occurred in PAL cluster (Fig. 7-E1, *p*<0.05, one-way ANOVA, Sidak multiple comparison test), PPL1 cluster (Fig. 7-E2, *p*<0.01, one-way ANOVA, Sidak multiple comparison test), and total clusters (Fig. 7-E6, *p*<0.05, one-way ANOVA, Sidak multiple comparison test). Notably, at 30 days old, not only the improvement in PAL and PPL1 cluster (Fig. 8-E1, *p*<0.05 and 8-E2, *p*<0.01 respectively, one-way ANOVA, Sidak multiple comparison test ) but the rescue also was further reported in PPM3 cluster (Fig. 8-E5, *p*<0.05, one-way ANOVA, Sidak multiple comparison test), which suggested the strong capacity of SFWE in the long-term treatment. On the other hand, SFWE at the concentration of 1.25 mg/mL did not impact the number of DA neurons in the control fly in the adult stage (Figs. 7, 8-E1-E5, *p*>0.05, one-way ANOVA, Sidak multiple comparison test). Besides that, similar to the previous study, the knockdown dUCH triggered the loss of DA neurons, which could be observed in the reduction of total DA neurons from day 1 to 30 and increased over time.

### Sim fruit water extract decreased ROS level induced by knockdown of dUCH

**Fig. 9:**
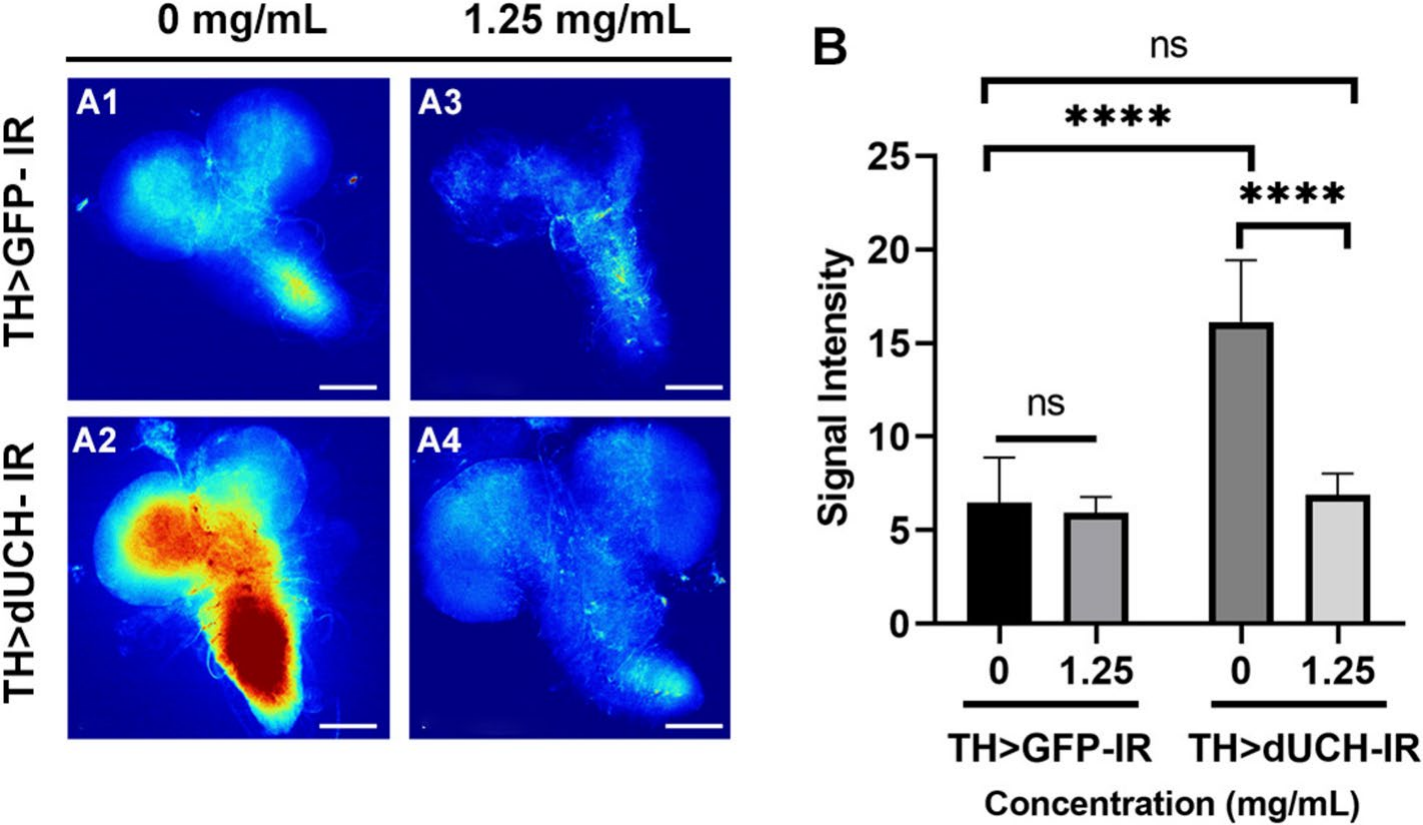
Sim fruit water extract rescued the level of ROS. A1-A4: The larvae brain lobes were stained with CM-H2DCFDA. B: The data described the intensity signal of ROS (the relative ROS). Sample size *n* = 4–6 flies per strain, one-way ANOVA, Sidak multiple comparison test. The control strain TH > GFP-IR (+ ; UAS-GFP-IR; TH-GAL4) and dUCH-knockdown strain TH > dUCH-IR (+ ; + ; TH-GAL4/UAS-dUCH-IR). Scale bars indicate 200 μm. ns: not significant, and ****p < 0.0001. Data represents means and the standard deviation (SD)

Oxidative stress is one of the main molecular mechanisms contributing to PD. The accumulation of ROS in the substantia nigra was reported in most Parkinson's patients [5]. The dUCH knockdown larvae were treated with SFWE and then evaluated reactive oxygen species (ROS) levels by 2’,7’ –dichlorofluorescein diacetate (also known as H_2_-DCFDA) in brain tissue. Experimental results showed that the intensity of fluorescence formed based on the association of ROS and H_2_-DCFDA in untreated dUCH knockdown larvae was significantly higher 2,48-time than in untreated control flies (Fig. 9-B, *p* < 0.0001, one-way ANOVA, Sidak multiple comparison test), which suggested the accumulation of ROS due to the dUCH deficiency. Interestingly, SFWE at 1.25 mg/mL concentration reduced the relative level of ROS in flies with gene defect (Fig. 9-B, *p* < 0.0001, one-way ANOVA, Sidak multiple comparison test), especially the signal intensity was equational level with the normal flies (Fig. 9-B, *p* > 0.05, one-way ANOVA, Sidak multiple comparison test). Besides that, the extract did not affect the control files (Fig. 9, *p* > 0.05, one-way ANOVA, Sidak multiple comparison tests).

## Discussion

*Parkinson’s disease* (PD) is a common neurodegenerative disease, identified by the loss of dopaminergic neurons (DA) and shortage of dopamine leading to abnormal mobility. Until now, the main reason contributing to the disease and thorough treatment is still unclarified. Several previous studies demonstrated the strong association between PD and oxidative stress, antioxidant compounds thus becoming a potential therapy. Recent studies focused on screening plants with high antioxidant properties for PD treatment, which helps diversify medicinal sources and limit the side effects when used for a long time. This study utilized the PD *Drosophila melanogaster* model following Hiep et.al research [16], by knocking down dUCH at specific DA neurons, to evaluate the therapy effectiveness of the *Rhodomyrtus* tomentosa fruit extract. Similar to the previous, our study showed that knocking down dUCH *Drosophila* model mimicked PD-like phenotypes including defects in locomotor and loss of DA neurons. Besides that, we also reported an increase in the level of ROS reflecting oxidative stress, which has not been previously observed in brain tissue.

Sim (*Rhodomyrtus* tomentosa) belongs to the *Myrtaceae* family, which has been used in traditional medicine for a long time. Different parts of sim could be used for the treatment of numerous diseases, among them berried sim often cures digestive and infectious disease [17, 21]. Sim fruit was the highest bioactive component due to containing 19 different types of phenolic compounds, the most notable were ellagitannin and stilbene derivatives. Among them, piceatannol was the highest contained in sim fruit [22]. The antioxidant capacity of sim fruit was also equivalent to other phenolic risk fruits such as strawberry, blackberry, and blueberry and higher than consumed fruit such as mango, kiwi, and apple [23]. In this study, the antioxidant ability was evaluated by DPPH assay, IC _50_ of SFWE and vitamin C were 55,55 ± 2.012 μg/mL and 11.34 ± 0.235 μM respectively (Fig. 1), which proposed 5 mg/mL of the extract would have equivalent effects with 1 μM vitamin C. Interestingly, the antioxidant capacity of SFWE evaluated by DPPH assay higher than other extracts being effective on PD treatment when comparing correlation with vitamin C [11, 12, 29]. With strong antioxidant capabilities, phenolic compounds may protect or reduce oxidative stress, a phenomenon commonly observed in Parkinson's patients. In addition, some phenolic compounds in sim fruit such as piceatannol, anthocyanin derivatives, and flavonol derivatives with anti-inflammatory abilities [30–33] may contribute to the mitigation of neuroinflammatory conditions, which is one of the underlying mechanisms requiring further investigation. Some recent studies also highlighted the effects of piceatannol, the most abundant phenolic compound in sim fruit, in neuroprotection by rescuing mitochondrial dysfunction [34] or inducing mitophagy and mitobiogenesis [35]. This finding further emphasizes the potential sim fruit as a novel source of antioxidant therapy for neurodegenerative diseases.

As expected, our results showed that sim fruit water extract (SFWE) at 1.25 mg/mL concentration could rescue PD-like phenotypes, caused by knockdown of dUCH. In detail, we noticed 20% augmentation in the number of DA neurons and 42.8% decrease in ROS levels, compared to the untreated defect larvae, which suggested early curative competency. Parkinson’s disease often occurs in older stages, however, the disease may progress silently in a young period. Our results highlight the rescue of cellular phenotypes via dUCH deficiency in the initial stage, which points out a change for early treatment and prevention. The improvement in DA neuron number was continuously reported in the adult stage from 1 to 30 days old, expressing the effectiveness of long-term treatment. Concrete, the degenerated percentage of DA neurons reduced from 13.4% to 6% when treated with the extract at 1.25 mg/mL. Not only cellular phenotype, but SFWE could also significantly increase adult flies' locomotor ability from 10 days old and continuously to 30 days old. Notably, the improvement percentage rose 25%, 38%, 42%, and 67% respectively from day 1 to 30. These results demonstrate that SFWE initially rescued cellular PD-like phenotypes, subsequently leading to the improvement of mobility symptoms. Besides that, the curative efficiency was observed at different stages, highlighting the potential for both early and late-stage treatment. Side effect associated with prolonged treatment was a critical issue that warrants careful consideration. Our data provided partial insights into this issue, utilizing SFWE at 5 mg/mL caused a decline in the mobility of normal flies at 20 days old and continuously at 30 days old, which suggested the overdose hoarding. By contrast, this phenomenon was not significantly observed in knockdown flies simultaneously because of the smaller quantity of excess compounds.

Besides the pharmaceutical activities, the impact of the extract on physiology was investigated. In general, sim fruit at all investigated concentrations did not affect the development time as well as the hatching rate of fruit flies in both strains (Fig. 2). However, high concentrations caused a shortened lifespan in dUCH knockdown flies (Fig. 3). Given the results, we speculate that SFWE at low concentrations was non-toxic to fruit flies; by contrast, the concentration higher than 5 mg/mL caused reduced lifespans in gene defect flies but not normal flies. This proposed the more vulnerable dUCH knockdown flies compared to the control, which was consistent with the results described above as well as previous studies [12, 13].

In addition, food intake partially expressed the nutritional content received, an important factor that needed to be investigated when utilizing oral treatment. Interestingly, SFWE caused an alternation of medium flavor leading to whet their appetite (Fig. 4). However, 1.25 mg/mL concentration, which was effective in PD treatment, did not cause a significant change in the food they received. These data once again supported the result that SFWE at suitable concentrations did not influence the physiology of fruit flies. In addition, the increase in food intake was observed in the normal files but not in dUCH knockdown flies, which suggested less sense of taste in the flies with gene defects.

Taken together, our results highlight the effectiveness of SFWE in delaying and improving PD-like phenotypes caused by the knockdown of dUCH and their safety effect in normal development when using a suitable dosage.

## Conclusions

Parkinson’s disease is a common degenerative disease, which strongly influences the life quality of patients. This study represents the first demonstration of the potential of sim fruit water extract to ameliorate the onset of PD-like phenotypes including locomotor dysfunction, and loss of DA neurons. Additionally, the elevation of oxidative stress in brain tissue due to dUCH deficiency was reported for the first time. This phenomenon was also rescued following the treatment of SFWE, which emphasized the therapeutic potential of antioxidants in the treatment of neurodegenerative disorders. Sim fruit water extract thus was a promising botanical source for the treatment of Parkinson's disease, applicable to both early and later stages.

## Supplementary Information

Additional file 1: Figure S1: Effect of knockdown dUCH on *Drosophila melanogaster* lifespans. *n*= 100, Log-rank test. The control strain TH>GFP-IR (+; UAS-GFP-IR; TH-GAL4) and dUCH-knockdown strain TH>dUCH-IR (+; +; TH-GAL4/UAS-dUCH-IR), *p<0.05. Figure S2: Sim fruit water extract rescued the loss of dopaminergic neurons (DA) caused by the knockdown of dUCH in adults 1 day old. A1: The data described the number of DA neurons in PAL clusters. A2: The data described the number of DA neurons in PPL1 clusters. A3: The data described the number of DA neurons in PPL2 clusters. A4: The data described the number of DA neurons in PPM1/2 clusters. A5: The data described the number of DA neurons in PPM3 clusters. A6: The data described the number of DA neurons in total clusters, *n*= 8-11, one-way ANOVA, Sidak multiple comparison test. The control strain TH>GFP-IR (+; UAS-GFP-IR; TH-GAL4) and dUCH-knockdown strain TH>dUCH-IR (+; +; TH-GAL4/UAS-dUCH-IR). ns: not significant, *p<0.05, and **p<0.01. Data represents means and the standard deviation (SD).

## Data Availability

The data and material will be available on request.

## Abbreviations

ANOVA: Analysis of variance
H2-DCFDA: 2’,7’ –Dichlorofluorescein diacetate
dUCH: Drosophila ubiquitin carboxyl-terminal hydrolase
DA: Dopaminergic neurons
DPPH: 1,1-Diphenyl-2-picrylhydrazyl
IC50: Half-maximal inhibitory concentration
NGS: Normal goat serum
ns: Not significant
OD: Optical density
PD: Parkinson’s disease
PBS: Phosphate buffered saline
PBS-T: Phosphate-buffered saline/Tween
PFA: Paraformaldehyde
ROS: Reactive oxygen species
SFWE: Sim fruit water extract
SN: Substantia nigra
SD: Standard deviation
SE: Standard error
TH: Tyrosine Hydroxylase
TH>GFP-IRTH: +; UAS-GFP-IR; TH-GAL4
TH>dUCH-IR: +; +; TH-GAL4/UAS-dUCH-IR
UCH-L1: Ubiquitin carboxyl-terminal hydrolase L1
USA: United States of America
