# Supplementary material for: Targeting the Parkinson’s disease using sim (Rhodomyrtus tomentosa) fruit water extract
Source: BMC Complement Med Ther. 2025 May 14;25:173. doi: 10.1186/s12906-025-04844-8 (PMC12076925; doi:10.1186/s12906-025-04844-8)
Supplement: Supplementary file 1 — Additional file 1: Figure S1: Effect of knockdown dUCH on Drosophila melanogaster lifespans. n= 100, Log-rank test. The control strain TH>GFP-IR (+; UAS-GFP-IR; TH-GAL4) and dUCH-knockdown strain TH>dUCH-IR (+; +; TH-GAL4/UAS-dUCH-IR), *p<0.05. Figure S2: Sim fruit water extract rescued the loss of dopaminergic neurons (DA) caused by the knockdown of dUCH in adults 1 day old. A1: The data described the number of DA neurons in PAL clusters. A2: The data described the number of DA neurons in PPL1 clusters. A3: The data described the number of DA neurons in PPL2 clusters. A4: The data described the number of DA neurons in PPM1/2 clusters. A5: The data described the number of DA neurons in PPM3 clusters. A6: The data described the number of DA neurons in total clusters, n= 8-11, one-way ANOVA, Sidak multiple comparison test. The control strain TH>GFP-IR (+; UAS-GFP-IR; TH-GAL4) and dUCH-knockdown strain TH>dUCH-IR (+; +; TH-GAL4/UAS-dUCH-IR). ns: not significant, *p<0.05, and **p<0.01. Data represents means and the standard deviation (SD). [file 12906_2025_4844_MOESM1_ESM.docx]

**Supplementary**

**
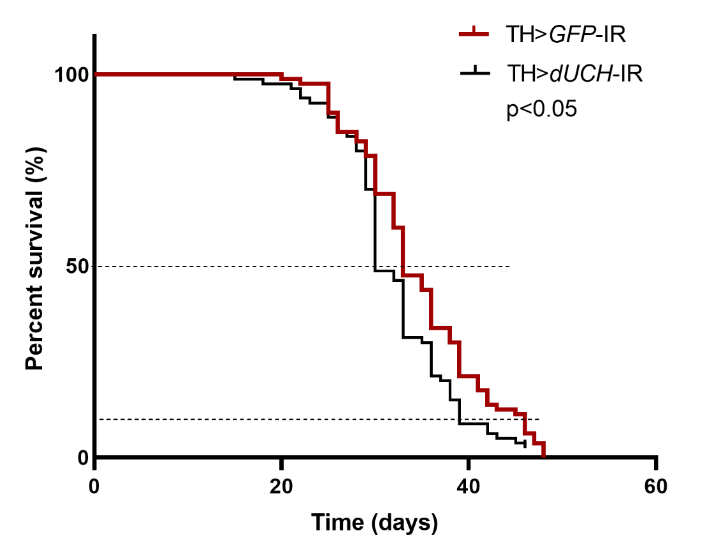
**

Figure S1: Effect of knockdown dUCH on *Drosophila melanogaster* lifespans. n= 100, Log-rank test. The control strain TH>GFP-IR (+; UAS-GFP-IR; TH-GAL4) and dUCH-knockdown strain TH>dUCH-IR (+; +; TH-GAL4/UAS-dUCH-IR), * p<0.05.


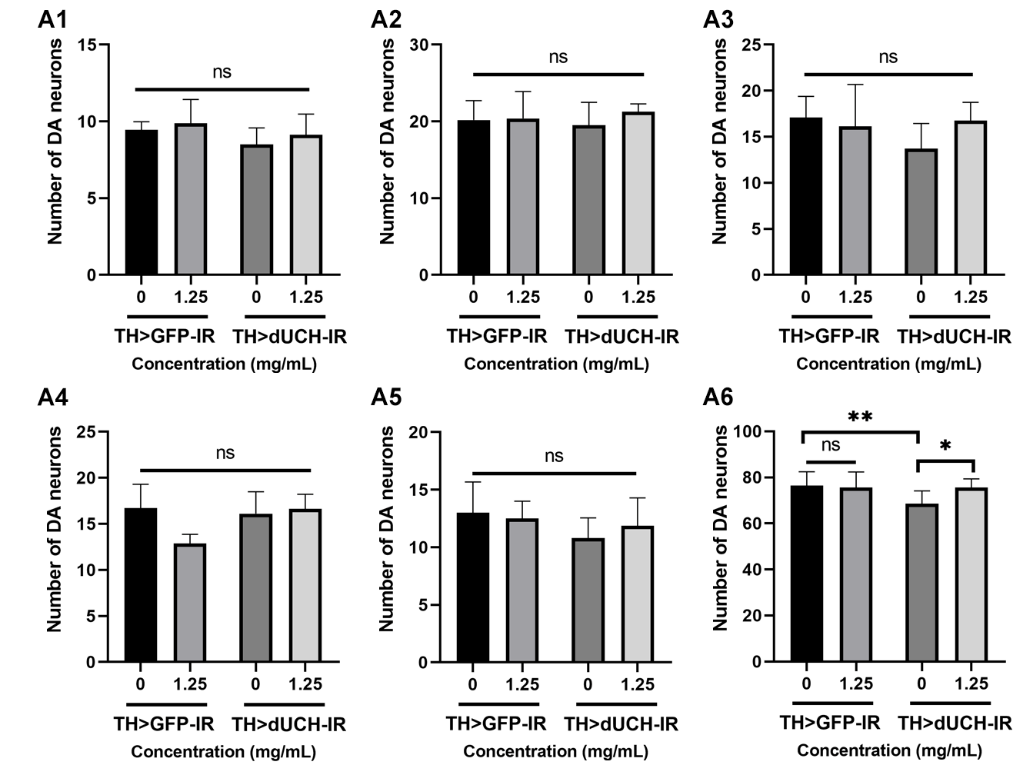


Figure S2: Sim fruit water extract rescued the loss of dopaminergic neurons (DA) caused by the knockdown of dUCH in adults 1 day old. A1: The data described the number of DA neurons in PAL clusters. A2: The data described the number of DA neurons in PPL1 clusters. A3: The data described the number of DA neurons in PPL2 clusters. A4: The data described the number of DA neurons in PPM1/2 clusters. A5: The data described the number of DA neurons in PPM3 clusters. A6: The data described the number of DA neurons in total clusters, n= 8-11, one-way ANOVA, Sidak multiple comparison test. The control strain TH>GFP-IR (+; UAS-GFP-IR; TH-GAL4) and dUCH-knockdown strain TH>dUCH-IR (+; +; TH-GAL4/UAS-dUCH-IR). ns: not significant, * p<0.05, and ** p<0.01. Data represents means and the standard deviation (SD).
